# The Rising Menace: Carbapenem-Resistant Klebsiella pneumoniae in a Tertiary Care Center and Co-dominance of blaNDM, blaOXA-48 Along With the Emergence of blaVIM

**DOI:** 10.7759/cureus.94303

**Published:** 2025-10-10

**Authors:** Fatima Muneer, Nikhil Raj, Anupam Das, Vikramjeet Singh, Manodeep Sen, Jyotsna Agarwal

**Affiliations:** 1 Microbiology, Dr. Ram Manohar Lohia Institute of Medical Sciences, Lucknow, IND; 2 Medical Microbiology, Sanjay Gandhi Post Graduate Institute of Medical Sciences, Lucknow, IND

**Keywords:** blandm, blaoxa-48, blavim, carbapenem resistance, klebsiella pneumoniae, mcim, mht, pcr

## Abstract

Background and objective

Carbapenem-resistant *Klebsiella pneumoniae* (CR-Kp) has become a significant cause of hospital-acquired infections, with significant implications for patient outcomes due to limited treatment options and high mortality rates. This study aimed to determine the prevalence, phenotypic resistance patterns, and molecular characteristics of CR-Kp isolates.

Methods

This was a cross-sectional study conducted for a period of one year. A total of 186 non-duplicate *Klebsiella pneumoniae (K. pneumoniae)* isolates from various samples were subjected to antibiotic susceptibility testing. Phenotypic detection of carbapenemase production was performed using the modified Hodge test (MHT), modified carbapenem inactivation method (mCIM), and combined disc testing (CDT). Genotypic analysis using PCR was performed on 20 representative CR-Kp isolates to detect *bla_NDM_, bla_KPC_, bla_OXA-48_, bla_IMP_, and bla_VIM_* genes.

Results

Out of 186 *K. pneumoniae* isolates, 120 (64.52%) were carbapenem-resistant based on Kirby-Bauer disc diffusion method. Respiratory and bloodstream infections showed the highest CR-Kp prevalence. Phenotypic methods detected carbapenemase activity in 61.29% (combined disk test), 48.92% (mCIM), and 40.86% (MHT) in *K. pneumoniae *isolates. Genotypic testing revealed *bla*_NDM_ in 100% and* bla*_OXA-48_ in 65% of the tested isolates, with *bla*_VIM_ detected in one isolate. No *bla*_KPC _or *bla*_IMP _genes were identified. CR-Kp isolates exhibited high resistance to fluoroquinolones, cephalosporins, and aminoglycosides.

Conclusions

The high prevalence of *bla*_NDM_- and *bla*_OXA-48_-mediated carbapenem resistance in *K. pneumoniae* highlights a growing threat to antimicrobial efficacy. Routine molecular surveillance and stringent antibiotic stewardship initiatives are urgently needed to limit the spread of CR-Kp in hospital settings.

## Introduction

*Klebsiella pneumoniae (K. pneumoniae)* is a major infectious agent implicated in both community-based and hospital-associated infections. In healthy people, *K. pneumoniae* is a normal component of the gastrointestinal microbiota; nevertheless, in some circumstances, especially in immunocompromised people or those with underlying medical disorders, it can become harmful [1,2]. Its ability to acquire multidrug resistance, particularly to carbapenems, has made it a significant public health concern. The main cause of *K. pneumoniae* pathogenicity includes certain virulence factors.. Lipopolysaccharide (LPS) on the bacterial cell wall plays a key role in immune evasion and inflammatory response modulation. Siderophores, iron-binding molecules, enable *K. pneumoniae* to access vital nutrients like iron from the host environment, aiding in its survival and proliferation [3,4].

The resistance of carbapenems is due to the expression of a carbapenemase enzyme, efflux pump, or loss of porin channels [5]. Carbapenem resistance in* K. pneumoniae *is primarily mediated through carbapenemase enzymes such as KPC, NDM, OXA-48, VIM, and IMP, leading to high mortality rates in infected patients. Multidrug-resistant (MDR) *K. pneumoniae *is a concerning matter in Indian hospitals due to the potential transfer of resistance to different pathogens [6]. The distressing pattern of rising resistance to carbapenems poses a major public health threat [7]. The abrupt emergence and subsequent outbreaks of NDM-1 likely serve as the primary example of the increased prominence of MBLs as a disease cause [8]. This study aimed to determine the prevalence, phenotypic resistance patterns, and molecular characteristics of Carbapenem-resistant *K. pneumoniae* (CR-Kp) isolates.

## Materials and methods

This was a cross-sectional study conducted over a period of one year. A total of 186 *K. pneumoniae* isolates were collected from urine, blood, respiratory samples, pus, and other body fluids of patients admitted in various wards, including the ICU and OPD. Blood culture bottles were incubated in the BacT/ALERT system and, once flagged positive, were inoculated on blood agar and MacConkey agar, followed by Gram staining of the isolates. Simultaneously, clinical samples were directly inoculated on MacConkey agar and blood agar and incubated aerobically at 37°C for 24-48 hours. Matrix-assisted laser desorption/ionization time-of-flight mass spectrometry (MALDI-TOF MS) was used to detect bacterial growth.

Antimicrobial susceptibility testing (AST) of *K. pneumoniae *isolates was performed using the Kirby-Bauer disc diffusion method on Mueller-Hinton agar plates. Isolates resistant to any carbapenem (ertapenem, imipenem, or meropenem) as per the latest Clinical and Laboratory Standards Institute (CLSI) guidelines were considered carbapenem-resistant and included for further analysis. 

**Figure 1:**
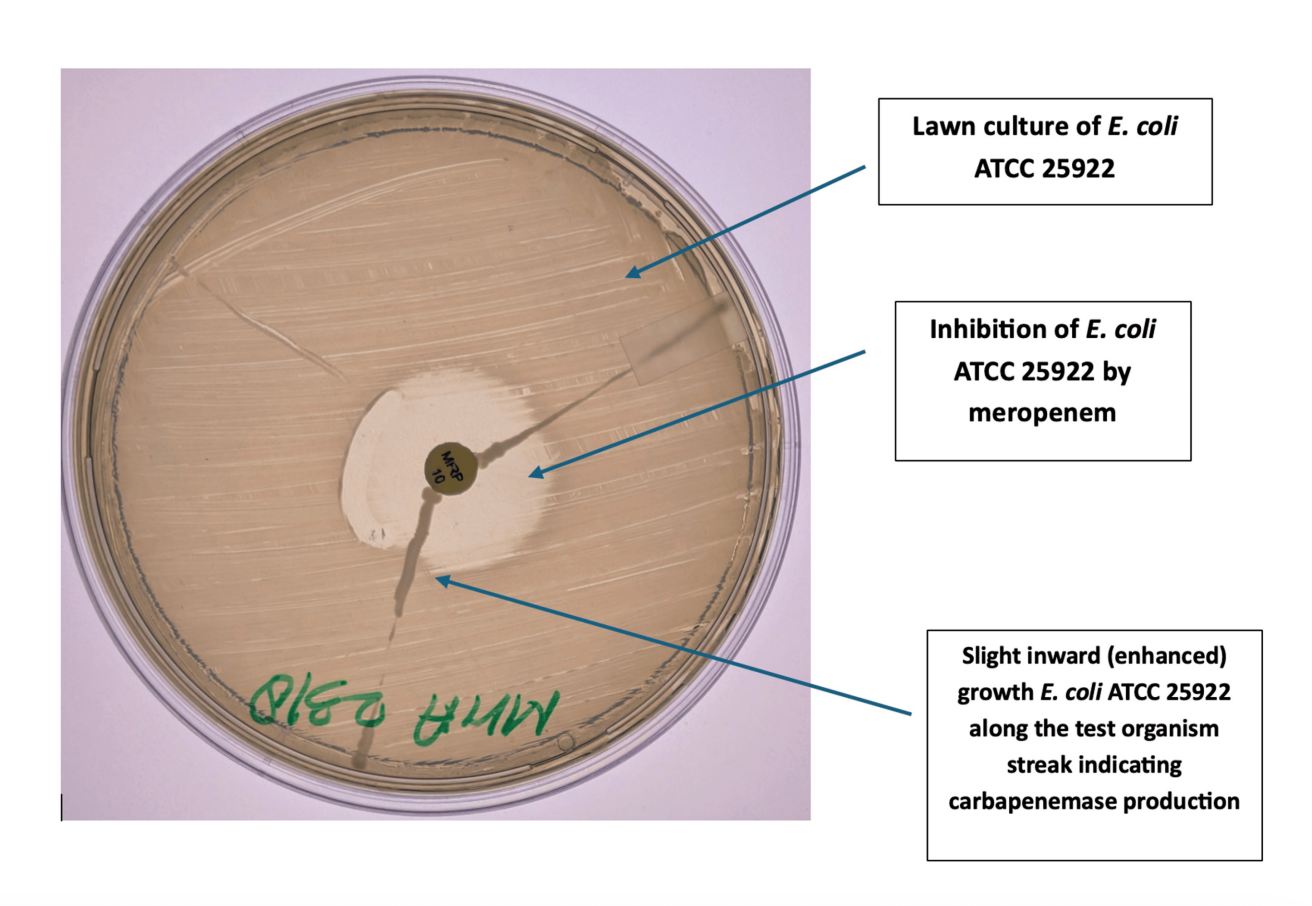
Modified Hodge Test (MHT)-positive Klebsiella pneumoniae isolate A lawn culture of* Escherichia coli (E. coli) *ATCC 25922 is streaked on Mueller–Hinton agar with a central meropenem (10 µg) disc. The test isolate of *Klebsiella pneumoniae *is streaked from the edge of the disc to the periphery. The characteristic cloverleaf-like indentation of *E. coli* growth along the streak indicates a positive MHT, confirming carbapenemase production

Metallo-β-lactamase (MBL) production was screened using the imipenem (10 μg)-EDTA (750 μg) combined disk method. Imipenem-only and imipenem-EDTA discs were positioned on the surface of a lawn culture of the test organism that had been produced on Mueller-Hinton agar. The difference in zone diameter of ≥7 mm between the imipenem-only and the imipenem + EDTA discs after 16-18 hours of incubation at 37°C denotes MBL production. The modified Hodge test (MHT) was used to further assess carbapenemase activity in accordance with CLSI M100-S19 (2009) recommendations. A meropenem (10 μg) disc was positioned in the center of a Mueller-Hinton agar lawn culture of *Escherichia coli* (*E. coli) *ATCC 25922, and the test strain was streaked from the disc's edge to the plate margin. A cloverleaf-like indentation of *E. coli* growth during an overnight incubation at 35 ± 2 °C showed a successful outcome, as shown in Figure 1.

The modified carbapenem inactivation method (mCIM) was performed by incubating a 10-µg meropenem disc in tryptic soy broth inoculated with the test strain for two to three hours at 37 °C. The disc was then transferred onto a Mueller-Hinton agar plate previously inoculated with *E. coli* ATCC 25922 (0.5 McFarland). After overnight incubation at 37°C, a zone of inhibition ≤15 mm indicated carbapenemase production; ≥19 mm suggested a negative result, while 16-18 mm or zones with colonies were considered indeterminate.

Genotypic detection of carbapenem resistance genes was performed using the TRUPCR Total Nucleic Acid Extraction Kit for DNA isolation from bacterial cultures, followed by real-time PCR with the TRUPCR Carbapenem Resistance Detection Kit. This assay qualitatively detected and differentiated key carbapenemase genes (*bla_KPC_, bla_NDM_, bla_VIM_, bla_OXA-48_, and bla_IMP_*) using extracted nucleic acid from BHI broth-inoculated isolates. 

Statistical analysis

Descriptive and inferential techniques were used in the statistical analysis. For the sample distribution, demographic factors, and patterns of antibiotic susceptibility, frequencies and percentages were computed. The relationship between sample type and carbapenem resistance was evaluated using the chi-square test; a p-value of less than 0.05 was deemed statistically significant.

## Results

Out of 186 *K. pneumoniae* isolates tested, 120 (64.52%) were found to be carbapenem-resistant based on the Kirby-Bauer disc diffusion method. The majority of infections were seen in male patients (55%) compared to females (45%). The age group of 41-60 years had the highest prevalence of CR-Kp at 38.71%, followed by the age group of 21-40 years at 29.56% (Table 1).

**Table 1:** Age and gender distribution of patients

Age group, years	Number of patients			Percentage
	Male	Female	Total	
0-20	9	8	17	9.13%
21-40	21	34	55	29.56%
41-60	42	30	72	38.71%
61-80	29	12	41	22.04%
81-90	1	0	1	0.53%
TOTAL	102	84	186	100%

The distribution of *K. pneumoniae* isolates among clinical specimens showed the highest number from urine samples (n = 86), of which 44 were carbapenem-resistant. Sputum and bronchoalveolar lavage (BAL) samples contributed 46 isolates with a high resistance rate (36/46), followed by blood (17/20). Statistical analysis revealed a significant association between sample type and carbapenem resistance in urine (p = 0.009) and central venous pressure (CVP) tip cultures (p = 0.046) (Table 2).

**Table 2:** Distribution of Klebsiella pneumoniae among different samples BAL: bronchoalveolar lavage; CVP: central venous pressure

Sample	No. of *Klebsiella pneumoniae* Isolates n (%)	No. of carbapenem-resistant* Klebsiella pneumoniae* n (%)	No. of carbapenem-sensitive* Klebsiella pneumoniae​​​​​*​​ n (%)	Chi-square test	P-value
Sputum and BAL	46 (24.7)	36 (30.0)	10 (15.1)	3.79	0.051
Blood	20 (10.8)	17 (14.2)	03 (4.5)	3.66	0.055
Urine	86 (46.2)	44 (36.7)	42 (63.6)	6.69	0.009
Body fluids	04 (2.2)	02 (1.7)	02 (3.0)	0.36	0.54
CVP	07 (3.8)	02 (1.7)	05 (7.6)	3.95	0.046
Pus	23 (12.4)	19 (15.8)	04 (6.1)	3.28	0.069

Among the phenotypic methods used for carbapenemase detection in CR-Kp isolates, the combined disk test (CDT) showed the highest sensitivity with 114 (61.29%) positive results, followed by the mCIM in 91 isolates (48.92%), and MHT in 76 isolates (40.86%) (Table 3).

**Table 3:** Phenotypic methods for detection of carbapenemase MHT: modified Hodge test; mCIM: modified carbapenem inactivation method; CDT: combined disc test

Carbapenemase detection method	Carbapenemase detected, n (%)	Carbapenemase not detected, n (%)
MHT	76 (40.86%)	110
mCIM test	91 (48.92%)	95
CDT	114 (61.29%)	72

**Figure 2:**
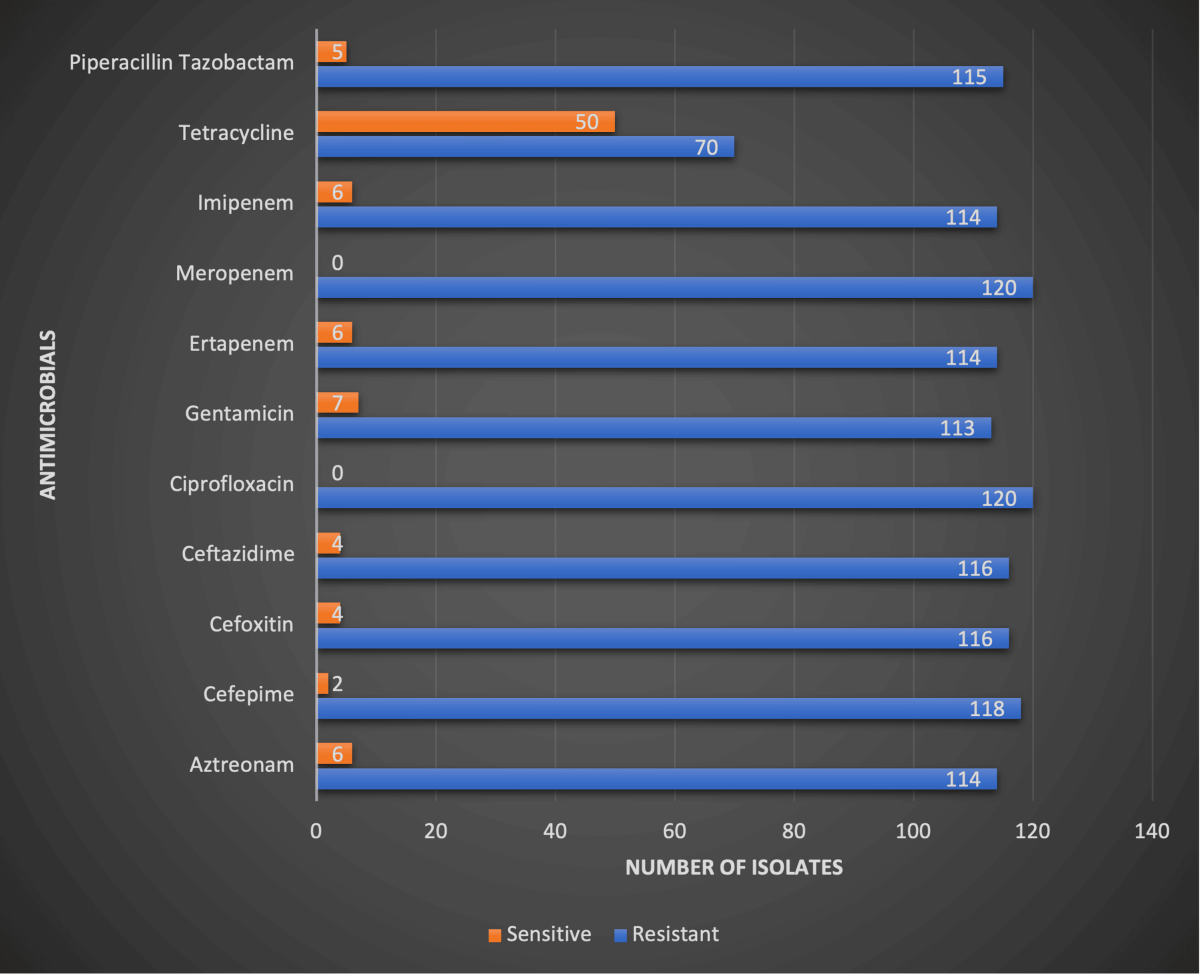
Antimicrobial susceptibility pattern of carbapenem-resistant Klebsiella pneumoniae isolates

Antibiotic susceptibility testing of the 120 CR-Kp isolates revealed high resistance rates across most antibiotics tested. All isolates were resistant to meropenem and ciprofloxacin. High levels of resistance were also seen with ceftazidime (96.7%), cefotaxime (96.7%), and aztreonam (95%). Only tetracycline showed moderate susceptibility, with 41.7% (50/120) of isolates being sensitive. The complete resistance profile is shown in Figure 2.

**Figure 3:**
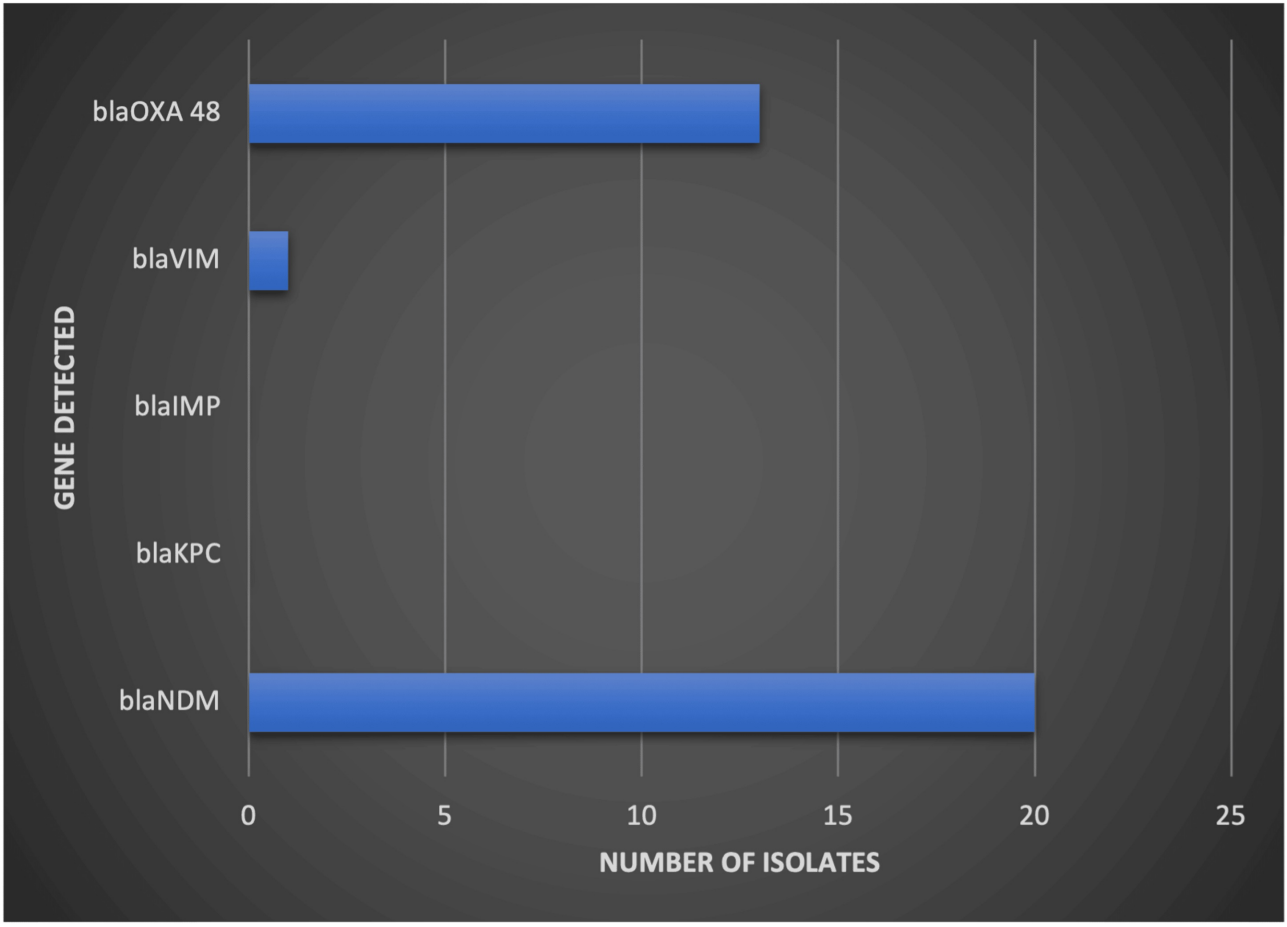
Distribution of carbapenemase genes among carbapenem-resistant Klebsiella pneumoniae isolates

Genotypic detection was performed on 20 CR-Kp isolates using real-time PCR. All 20 isolates (100%) were positive for the *bla_NDM_ *gene, while* bla _OXA-48_ *was detected in 13 isolates (65%). Additionally, *bla_VIM_* was found in only one isolate (5%), whereas *bla*_*KPC* _and *bla_IMP_ *were not detected in any of the tested isolates. The distribution of these genes is presented in Figure 3. 

## Discussion

The results of this study demonstrate a high prevalence of carbapenem resistance (120/186; 64.52%) among *K. pneumoniae *isolates, highlighting a significant concern for nosocomial infections and antimicrobial resistance management. This finding is consistent with regional data, where a systematic review reported the highest prevalence of CR-Kp in South Asia at 66.04%, with India specifically reporting 67.62% [9]. 

Indrajith et al. reported carabapenem resistance in 58% of *K. pneumoniae* isolates [10]. The observed resistance rate also aligns with recent studies from India, the Middle East, and Southern Europe, where co-production of *bla_OXA-48_* and *bla_NDM_* has been frequently reported. These results highlight the critical necessity for strict infection control protocols and strong antibiotic stewardship in order to stop the development of resistant bacteria in healthcare environments. The overall prevalence of carbapenem resistance in *Enterobacteriaceae* was found to be 29.35% in a study by Srivastava et al. [11]. 

In this study, sputum and BAL samples (46 isolates) showed a high carbapenem resistance rate of 78.2% (36 CR-Kp isolates), indicating a significant role of CR-Kp in ventilator-associated pneumonia (VAP) and respiratory tract infections (RTIs). Bloodstream infections (BSIs) demonstrated the highest resistance, with 85% (17 out of 20 cases) of isolates being CR-Kp, highlighting their impact in sepsis at our institute. Demographic data showed that males (55%) had a higher infection rate than females (45%), consistent with previous findings on* K. pneumoniae *infections. Abbasi et al. have similarly reported that 67.7% of cases were males and 32.2% were females [12]. In our study, phenotypic detection of carbapenemase production was performed using MHT, CDT, and mCIM, which detected carbapenemase activity in 76 (40.86%), 114 (61.29%), and 91 (48.92%) isolates, respectively. Similar observations were reported in the study by Manandhar et al., where CDT was described as the most sensitive method for MBL detection [13].

Our PCR analysis revealed that all tested CR-Kp isolates (n=20) harbored the *bla_NDM _*gene (100%), with 65% also carrying *bla_OXA-48_*, and one isolate (5%) harboring *bla_VIM_*, while *bla_IMP_* was not detected. These findings are consistent with previous Indian studies identifying *bla_NDM_ *and *bla*_*OXA-48* _as predominant carbapenemase genes [14-16]. A study in India reported a similar dominance of* bla_NDM_,* though *bla_OXA-48_* was less prevalent at that time [13]. Nordmann et al. have highlighted the global dissemination of *bla_NDM_ *and *bla_OXA-48_*, particularly in nosocomial outbreaks [16]. The detection of *bla_VIM_*, although rare, signals the emergence of Verona integron-encoded metallo-β-lactamase, known for broad β-lactam hydrolysis [16]. A prior study from our center reported NDM in 100% of carbapenem-resistant urinary isolates, followed by KPC (75%) and OXA-48 (60%), with no VIM detected [14]. Since *bla_NDM _*and *bla_OXA-48_* are linked to substantial drug resistance (XDR) and strong transmission potential, their confluence in 65% of isolates is especially concerning.

NDM-positive *K. pneumoniae* is globally recognized for high-level resistance and widespread dissemination [9,10]. While *bla_OXA-48_* confers carbapenem resistance, it often retains susceptibility to cefepime and aztreonam, complicating treatment [17]. The absence of *bla_IMP_*, typically reported in East Asia and Europe, further defines the regional resistance gene profile [18]. In a study by Mokhtari et al., the percentage of isolates with the carbapenemase expressing the *bla_ OXA-48_* and *bla _NDM-1 _*genes was 96.7% and 66.7%, respectively [19]. These results align with global data, reinforcing the challenge of managing CR-Kp infections and the critical importance of robust surveillance and stewardship strategies.

Limitations

This study was limited by genotypic testing of only a small subset of isolates, restricting full representation of resistance mechanisms. Being a single-center cross-sectional study, the results may not be generalizable. In addition, clinical outcomes were not evaluated, preventing a correlation between resistance patterns and patient prognosis.

## Conclusions

The high prevalence and antibiotic resistance of *Klebsiella pneumoniae *make it a serious challenge to hospital infection management. Effective surveillance, antimicrobial stewardship programs, and alternative treatment strategies are crucial to managing CR-Kp infections and preventing further dissemination.
